# Positive Effects of Caffeine Therapy in a Girl with PDE2A‐Related Paroxysmal Dyskinesia

**DOI:** 10.1002/mdc3.70019

**Published:** 2025-03-03

**Authors:** Katerina Bernardi, Moritz Thiel, Anne Koy

**Affiliations:** ^1^ Department of Pediatrics Faculty of Medicine and University Hospital of Cologne, University of Cologne Cologne Germany; ^2^ Department Human Neuroscience Sapienza University Rome Italy; ^3^ Center for Rare Diseases, Faculty of Medicine and University Hospital Cologne University of Cologne Cologne Germany

**Keywords:** caffeine, paroxysmal dyskinesia, PDE2A, movement disorders

Paroxysmal dyskinesias (PxD) are a heterogeneous group of rare conditions characterized by recurrent episodes of abnormal involuntary movements, lasting seconds to hours. They can present with ballism, choreoathetosis, myoclonus, dystonia, or other hyperkinetic movement disorders (MD), either isolated or combined. PxD can be inherited or acquired, often appearing in patients with normal neurological conditions between episodes.^1^ Since its first description in 2018, mutations in the cyclic nucleotide phosphodiesterase 2A (PDE2A) gene have been associated with an ultra‐rare condition characterized by PxD with developmental impairment and sometimes chronic permanent MD and epilepsy. PDE2A encodes a phosphodiesterase that degrades cyclic nucleotides, which act as intracellular second messengers for many signal transduction pathways in the central nervous system.^2^ To date, only biallelic changes were identified as disease‐causing variants in PDE2A, with merely 12 patients and six different variants described.^3^, ^4^, ^5^, ^6^ PDE2A‐related PxD typically presents with attacks of hyperkinetic movements comprising dystonia and choreoathetosis, which are usually pharmacoresistant.^3^, ^4^

We report a new case of PDE2A‐related dyskinesia with positive response to caffeine treatment.

The case involves a girl born to healthy, consanguineous parents, with healthy siblings and no family history of neurological diseases. Pregnancy and postnatal adaptation were uncomplicated. She exhibited global developmental delay, achieving independent walking at 2.5 years, with deficits in gross and fine motor skills and language development. At age five years, she experienced her first episodes of sudden muscle tone increase, involuntary rotating limb movements, falls, and fixed gaze, lasting approximately 30 seconds. These episodes increased in frequency up to 10 times daily. A cranial‐magnetic resonance imaging was normal and initial suspicion of epilepsy led to anti‐seizure medication with levetiracetam.

Short stature (<1st percentile) was documented with delayed bone growth (bone age 4.5 years at chronological age 6.8 years). Growth hormone deficiency, hypothyroidism, and celiac disease were excluded.

As the frequency of hyperkinetic episodes increased and anti‐seizure medication proved ineffective, a 24‐hour‐electroencephalography‐monitoring was conducted, revealing no epileptic discharges during the events and suggesting PxD. Nonetheless, oxcarbazepine was initiated.

At age nine, she was referred to our Pediatric Movement Disorders Unit. At that time, she experienced frequent daily episodes (5–6 per hour) of sudden falls because of twisting movements and dystonic posturing (Video 1). Episodes were absent during sleep and potentially triggered by stress, emotions, and febrile infections. Between these episodes, she did not show any hyperkinetic movements. A protective helmet was introduced because of fall‐related injury risks. She walked independently, but only spoke an incomprehensible‐fantasy language. Comparative genomic hybridization‐array and genetic panel analyses for PxD provided findings of unclear significance (aa(hg19)(1‐22,X)x2 and ATP1A3 c.1620G>A;p = het AD). Ultimately, trio‐exome sequencing revealed a homozygous pathogenic variant in the PDE2A gene (Chr.11,c.1922+5G>A), already previously reported.^4^

**VIDEO 1:** Clinical condition before caffeine therapy. Girl, 9 years. Paroxysmal episode characterized by twisting movements and dystonic posturing associated with sudden falls.

After unsuccessful therapeutic attempts with oxcarbazepine, flunarizine, and phenytoin, a trial with caffeine was initiated. She experienced significant benefits, with reduction in frequency and intensity of hyperkinetic episodes, occasionally achieving completely symptom‐free days. The initial caffeine titration temporarily led to some prolonged hyperkinetic attacks and difficulty falling asleep. After overcoming these initial side effects, she tolerated the medication well without behavioral changes or digestive issues. The dose was gradually increased, with progressive reduction of hyperkinetic episodes. On reaching a daily dosage of 600 mg (3 × 200 mg caffeine tablets), only brief episodes of motor restlessness persisted upon awakening, spontaneously resolving after 30 minutes. Later, during a febrile episode, increased hyperkinetic movements, primarily oro‐buccal, were reported, despite unchanged ongoing caffeine therapy. However, post‐fever resolution led to gradual recovery, and she returned to stable clinical condition.

The hyperkinetic movements of our patients clearly improved with caffeine. Caffeine is known to be an antagonist of adenosine A2 (A2A) receptors and the modulation of both A2A and dopamine D2 receptors in the striatum plays a pivotal role in the inhibitory control of movement through the striatopallidal neurons of the indirect pathway.^7^

Caffeine has already been used in patients with ADCY5‐related dyskinesia, showing overall positive results and good tolerance even in children.^8^

Its potential role as an A2A receptor antagonist in controlling dyskinesia was also documented in a Caenorhabditis elegans model harboring two common GNAO1 mutations.^9^ GNAO1, ADCY5, and PDE2A genes encode proteins involved in G‐protein‐coupled receptor (GPCR) signal transduction and the regulation of the striatal cyclic adenosine monophosphate (cAMP) level.^10^ A2A antagonism in striatopallidal neurons would reduce Galphaolf‐mediated AC5 activation and the consequent cAMP synthesis, counteracting the decreased cAMP degradation related to PDE2A deficiency.

In our case, caffeine led to a great reduction of the frequency and intensity of hyperkinetic episodes, resulting in significant improvement in quality of life for the patient by increased autonomy in daily activities, reduced risk of falls and injuries, and improved ability to participate in the patient's social environment. The dosage of caffeine for patients with ADCY5 ranges between 60 and 800 mg/day.^8^ In our patient, high dosages (20 mg/kg) led to significant improvement. Furthermore, it should be considered to increase the dosage of caffeine or to add clonazepam during febrile episodes to avoid exacerbations of the MD. Prolonged follow‐up and further scientific data are necessary to determine the long‐term effects of caffeine in these patients. Nonetheless, we suggest caffeine as a potential treatment in patients with PDE2A associated paroxysmal dyskinesias in the absence of other efficacious therapeutic options.

## Author Roles

(1) Research project: A. Conception, B. Organization, C. Execution; (2) Statistical Analysis: A. Design, B. Execution, C. Review and Critique; (3) Manuscript Preparation: A. Writing of the First Draft, B. Review and Critique;

K.B.: 1A, 1B, 1C, 3A.

M.T.: 1A, 3B.

A.K.: 1A, 3B.

## Disclosures

**Ethical Compliance Statement:** Institutional review board approval was not required for this case report. A written informed consent was obtained from the patient's parents for this case report and its publication. We confirm that we have read the Journal's position on issues involved in ethical publication and affirm that this work is consistent with those guidelines.

**Funding Sources and Conflict of Interest:** No specific funding was received for this work. The authors declare that there are no conflicts of interest relevant to this work.

**Financial Disclosures for the Previous 12 Months:** A.K. receives grants by the Dr. Hans‐Günther and Dr. Rita Herfort foundation and the University of Cologne. She was the PI of the STIM‐CP‐trial, partly funded by Boston Scientific.

## Data Availability

The data that support the findings of this study are available from the corresponding author upon reasonable request.
